# Which one? A comparative study of traditional and sports uniforms on academic achievement, cognitive performance, playtime, bullying, and discrimination in adolescents: The Cogni-Action Project

**DOI:** 10.3389/fpubh.2022.917970

**Published:** 2022-08-12

**Authors:** Carlos Cristi-Montero, Patricio Solis-Urra, Javier Sanchez-Martinez, Jorge Olivares-Arancibia, Sam Hernández-Jaña, Guillermo Gajardo-Araya, Ximena Palma-Leal, Kabir P. Sadarangani, Matias Portela Estinto, Yonatan Encina, Cristian Alvarez, Pedro Delgado-Floody, Nicolas Aguilar-Farias, Gerson Ferrari, Sandra Mahecha-Matsudo, Juan Pablo Zavala-Crichton, Jessica Ibarra-Mora, Maribel Parra-Saldías, Rodrigo Nanjarí-Miranda, Fernando Rodríguez-Rodríguez

**Affiliations:** ^1^IRyS Group, Physical Education School, Pontificia Universidad Católica de Valparaíso, Valparaíso, Chile; ^2^Department of Physical and Sports Education, Faculty of Sport Sciences, PROFITH “PROmoting FITness and Health Through Physical Activity” Research Group, Sport and Health University Research Institute (iMUDS), University of Granada, Granada, Spain; ^3^Nuclear Medicine Services, “Virgen de Las Nieves” University Hospital, Granada, Spain; ^4^Faculty of Education and Social Sciences, Universidad Andres Bello, Santiago, Chile; ^5^Escuela de Kinesiología, Facultad de Salud, Universidad Santo Tomás, Viña del Mar, Chile; ^6^Grupo AFySE, Investigación en Actividad Física y Salud Escolar, Escuela de Pedagogía en Educación Física, Facultad de Educación, Universidad de las Américas, Santiago, Chile; ^7^Magíster en Educación, Mención Política y Gestión Educativa, Facultad de Filosofía y Humanidades, Universidad Austral de Chile, Valdivia, Chile; ^8^Escuela de Kinesiología, Facultad de Odontología y Ciencias de la Rehabilitación, Universidad San Sebastián, Providencia, Chile; ^9^Escuela de Kinesiología, Facultad de Salud y Odontología, Universidad Diego Portales, Santiago, Chile; ^10^Division of Healthy Public Policies and Promotion, Department of Health Promotion and Citizen Participation, Ministry of Health, Santiago, Chile; ^11^Facultad de Psicología, Universidad de Talca, Talca, Chile; ^12^Exercise and Rehabilitation Sciences Laboratory, Faculty of Rehabilitation Sciences, School of Physical Therapy, Universidad Andres Bello, Santiago, Chile; ^13^Department of Physical Education, Sports and Recreation, Universidad de La Frontera, Temuco, Chile; ^14^Universidad de Santiago de Chile (USACH), Escuela de Ciencias de la Actividad Física, el Deporte y la Salud, Santiago, Chile; ^15^Center of Studies on Physical Activity, Exercise and Health, Universidad Mayor, Santiago, Chile; ^16^Academic Unit, MEDS Clinic, Santiago, Chile; ^17^Dpto. de Educación Física, Deportes y Recreación, Universidad Metropolitana de Ciencias de la Educación, Santiago, Chile; ^18^Department of Physical Education, University of Atacama, Copiapó, Chile

**Keywords:** education, health, students, policy, mental health, physical activity

## Abstract

**Objective:**

The aim of this study was to compare academic achievement, cognitive performance, playtime, bullying, and discrimination in adolescents according to traditional uniforms (TUs) and sports uniforms (SUs) worn at school, while simultaneously exploring the influence of the school vulnerability index.

**Methods:**

A total of 988 Chilean adolescents (52.6% boys) aged 10–14 years participated in this cross-sectional study. Academic achievement was evaluated by the average grade in maths, language, and science grades, while cognitive performance was assessed through eight cognitive tasks. TUs affecting physical activity, playtime, bullying, and discrimination were queried. Mixed model analyses were performed.

**Results:**

No differences were observed in academic achievement (TU: 5.4 ± 0.1 vs. SU: 5.5 ± 0.2, p = 0.785) or in cognitive performance (TU: 99.6 ± 0.8 vs. SU: 98.9 ± 1.8, p= 0.754) according to the school uniformtype. Moreover, 64.1 % of participants declared that wearing TU affects their physical activity (traditional uniforms: + 8 min and sports uniforms: + 20 min), and those who believed so spent more time playing than those who answered negatively (14.5 min, p = 0.012). Finally, adolescents wearing SU displayed a lower feeling of bullying and discrimination; this finding depended mainly on the school's vulnerability.

**Conclusion:**

It is concluded that wearing TU does not show an educational advantage at an academic and cognitive level that justifies its obligation. In addition, it could be suggested that schools consider adolescents' opinions in adopting a more comfortable uniform, such as the SU. This feasible and low-cost measure would help to increase adolescents' physical activity during the school day, and, contrary to belief, it would not be related to increased feelings of bullying and discrimination.

## Introduction

School uniforms have an important social context; in fact, in New Zealand, it is a reason why students choose their schools (1), but in general, uniforms have been adopted as strategies by principals and governments to improve educational test scores and student behavior (2, 3). Indeed, several studies have suggested that traditional uniforms remove social differences, increase security, reduce violence, improve school climate and discipline, promote social responsibility, and improve academic achievement (2–8). However, findings on this matter are highly sparse and heterogeneous and seem to be dependent on the population studied, age, race, educational context, and socioeconomic status (2, 3, 6, 9, 10), and the aforementioned features have not been proven yet (3, 6, 7, 9). Given that most evidence about this research issue comes from developed countries, ascertaining uniforms' influence in a less advantageous social context (e.g., in a Latin-American country such as Chile) could give us more insight into this public matter.

In Chile, most schools require wearing a traditional uniform (TU) (i.e., shirt and school necktie in boys and skirt and blouse in girls), and gradually, some schools have begun to encourage wearing a sports uniform (SU) (i.e., polo shirts or t-shirts and sport or short trousers). A plausible reason is that, due to COVID-19, many schools have performed their classes virtually, and TUs have not been mandatory; thus, many schools and families have begun to question its use (i.e., economic concern). Uniforms are expensive, and most schools require that families buy several uniforms (i.e., for winter, summer, sports, and formal occasions), increasing households' economic burden considerably (8, 11, 12). Furthermore, in Chile, not wearing a TU has been used as a reason for school expulsion, contravening current national regulations (13).

In a highly unequal and hierarchical community such as Latin-American countries (14), promoting freedom of expression and reducing social gaps such as gender is a priority (15). However, wearing TU could limit self-expression (12) and could reduce engaging in physical activity, especially in girls (16), which further widens the gender gap at an early stage (17, 18), increasing health inequalities (19) and affecting cognitive and educational goals (20).

In this sense, family socioeconomic status is strongly related to adolescents' academic and cognitive performance; nonetheless, Latin-American schools seem to be stronger predictors of these indicators because they share certain features (i.e., economic, social, and cultural status) eliciting a more considerable influence than families (21). Indeed, a study on Chilean adolescents showed that a potent socioeconomic indicator at the school level (i.e., the school vulnerability index [SVI]) was negatively related to physical fitness and cognitive performance (22). Moreover, economic, social, and cultural status is related to the school climate (23), promoting greater learning and reducing bullying and conflicts among students (24). Despite the abovementioned, there is a lack of studies exploring differences between TU and SU and their effect on academic, cognitive, and physical activities and psychological indicators such as bullying and discrimination feelings in the same student sample (6).

We propose to study all these relevant indicators, first, in order to improve the understanding of these concerns, which could help generate social and educational policy, especially in vulnerable communities (6), and second, in contexts with greater social inequality, to demonstrate that possible differences due to the type of school uniform on outcomes would disappear when the socioeconomic status is considered. Therefore, this study establishes to determine whether adolescents wearing TUs present differences from those wearing SUs in (a) educational achievements (i.e., academic achievement and cognitive performance), (b) playtime, and (c) bullying and discrimination at school. Finally, we carried out two statistical models with and without the SVI, to determine whether possible differences between groups depend mainly on this proxy of a socioeconomic indicator at the school level.

## Methods

### Study design and ethical requirements

This cross-sectional study is part of the Cogni-Action Project carried out from March 2017 to October 2019 (25). The project was conducted according to the guidelines of the Declaration of Helsinki and approved by the Bioethics and Biosafety Committee of the Ethics Committee of Pontificia Universidad Católica de Valparaíso (BIOEPUCV-H103–2016). Written consents were obtained before participation from the school principal, parents, and participants.

### Participants

A total of 1,296 adolescents between the ages of 10 and 14 years, from 19 public, subsidized (i.e., schools that receive partially governmental and private economic support), and private schools of the Valparaiso region (Chile) participated in this study. From the 1,296 participants, a total of 308 adolescents were removed because their school (k = 4) wore a mixture of uniforms (a detailed explanation in the next section). Thus, a total of 988 adolescents participated in this study from 15 schools (k = 12 schools wearing traditional uniforms and k = 3 schools wearing sports uniforms).

### School uniform type

Traditional uniforms: Schools that declare their students wear a TU all weekdays except for physical education classes in which adolescents wear SUs. A TU for male adolescents was outlined as a polo shirt or shirt (with school necktie), sweater or blazer, and trousers, and for girls, a skirt and blouse, and sweater or blazer, both with school shoes (usually black leather).

Sports uniforms: Schools that declare their students wear SUs every day. An SU consisted of adolescents (both boys and girls) wearing mainly sportswear such as polo shirts or t-shirts and sport or short trousers (jeans were included as well in this category due to their generalized wearing), and sneakers.

### Educational achievements: Academic achievement and cognitive performance

Academic achievement was established by asking students their general average of the last semester in language, mathematics, and science and they were averaged. Grades in Chile are scored between 1 (minimum) and 7 (maximum) in which a grade of four implies approval. These three subjects are part of the Program for International Student Assessment.

The adolescents' cognitive performance was evaluated through eight neurocognitive tasks from the NeuroCognitive Performance Test (NCPT) from Lumos Labs, Inc. (26). This battery included “Trail Making A and B” assessing attention, cognitive flexibility, and processing speed; the “Forward Memory Span” and the “Reverse Memory Span” evaluating short-term visual memory and working memory; the “Go/No-Go” test assessing inhibitory control and processing speed; the “Balance Scale” indicating quantitative and analogical reasoning; the “Digit Symbol Coding” valuing processing speed; and finally, the “Progressive Matrices” assessing problem-solving and reasoning/intelligence (26). Each test was scaled following a normal inverse transformation of the percentile rank and summed obtaining a global cognitive score (27).

### Playtime

Playtime was estimated by the Chilean physical activity questionnaire validated in the population aged 8–13 years (28). Adolescents were asked about several physical activities. For this study, only a playtime item was considered. This item asks about the daily play (hours and min) in activities such as riding a bicycle, ball games, and running.

### Perception of traditional uniforms affecting physical activity

Adolescents were asked about the daily influence of TUs on physical activity levels. They respond “yes” or “no” to the following question: Do you think that wearing a TU in school affects your physical activity? The interviewers explained that the question refers to a decrease in the levels of physical activity during the school day.

### Suffered bullying and discrimination

Based on the questionnaire “Validation of a daily stress scale in Chilean schoolchildren” (29), we extracted two questions: (1) Have you suffered bullying in the classroom or in the schoolyard? and (2) Have you suffered discrimination in the classroom or the school playground? Each question scores from 1 to 4 concerning the frequency of the event (never: 1, sometimes: 2, usually: 3, and always: 4).

### Covariates

Six covariates (age, sex, maturation, physical fitness, BMI, and SVI) were included in the analyses because of their relevance and relationship with outcomes. It has been stated that sex and maturation are relevant factors associated with behaviors, cognitive, and brain development (30). The maturation was calculated according to the peak height velocity (PHV), subtracting the PHV age from the chronological age. Differences among years were established as a maturity offset value (31).

Physical fitness was evaluated by the ALPHA-fitness test battery (32), which comprises the assessment of cardiorespiratory fitness, muscular fitness, and speed-agility fitness. A *z*-score of each component was calculated and adjusted for age and sex, and all three were added (Global Fitness Score). Procedures and methodological details were published previously (27).

The height and weight were measured with a digital scale OMRON (HN-289-LA, Kyoto, Japan) with a precision of 0.1 kg and a portable stadiometer SECA (model 213, GmbH, Germany) with a precision of 0.1 cm, respectively. The World Health Organization 2007 growth reference was used to determine the body mass index expressed in z-score (BMI_Z_) for school-age children (33).

Additionally, SVI was included as a proxy of a socioeconomic factor at the school level (model 2). This Chilean index measures the degree of socioeconomic vulnerability of pupils who attend schools with partial or total state funding (subsidized and public schools, respectively) based on the educational level of parents-tutors, student health condition, physical and emotional wellbeing, and school location. This index score ranges from 0 to 100, with a score of zero being assigned to the private schools, and thus, schools were classified as low (<10), middle (≥10 to <60), and high (≥60) (20).

### Statistical analysis

Before analyses, data were imputed based on a nonparametric missing value method using random forest through the “missForest” R package (34). This function successfully imputes large and complex mixed-type datasets (quantitative and/or categorical variables), including complex interactions and nonlinear relations by a random forest trained on the observed values predicting the missing values. Missing data ranges were between 1.0 and 27.0%, and the estimation error was 0.081 (numeric variables) and 0.082 (factor variables). The measure with the highest number of imputations was the Global Fitness Score (a covariate). The central limit theorem for sample sizes over 500 participants was considered (35). A Q-Q plot (quantile-quantile plot) was used for checking normality visually. Additionally, we explored a possible interaction between sex and age. No interaction was observed; thus, analyses were not stratified by sex or age. Differences between groups were tested (*t*-test or chi-square according to variable features).

Mixed model analyses were performed to establish differences between TUs and SUs in the academic and cognitive performance, between those who respond that TUs affect their physical activity level (yes/no) at playtime, and finally at bullying and discrimination feelings. To compare the likelihood of a model with the effect included vs. a model with the effect excluded, the likelihood-ratio test (LTR) for the random effect was estimated. A significant value indicates that the model with random effect is significantly better (in terms of likelihood) than the model without the cluster, and the interclass correlation coefficient (ICC) was estimated. *Post-hoc* tests were estimated using the Holm correction to multiple comparisons. For all analyses, significant values were established at *p* < 0.05. All models were adjusted for multiple covariates. Model 1 included sex, PHV, BMIz, and physical fitness, while model 2 added the SVI in order to test the study hypothesis. Adolescent schools (k = 15) were used as clusters (random effect). All statistical analyses were conducted using the general, mixed and generalized models. We presented plots to simplify the main findings; however, complete tables with both models are presented as Supplementary material. The proportion test (2 outcomes) was used to establish differences in frequency response (yes/no) to the question: “Do you think that wearing a traditional uniform in school affects your physical activity?”. Moreover, contingency tables and chi-square tests were carried out to describe and corroborate, respectively, differences between answer categories for bullying and discrimination. All analyses were performed using the statistical software Jamovi version 2.0.0.0 (The Jamovi Project) (36).

## Results

Table 1 shows the descriptive characteristics of the sample. In this study, a total of 988 adolescents participated; of them, 854 (86.4%) participants wore a TU (k = 12 schools) and 134 (13.6%) participants wore a SU (k = 3 schools). Schools where adolescents wear TUs were public (k = 7), subsidized (k = 4), and private (k = 1), and nine presented high-SVI, two middle-SVI, and one low-SVI, while schools where adolescents wear SUs were subsidized (k = 2) and private (k = 1), and two presented middle-SVI and one low-SVI.

**Table 1:** Descriptive participant's characteristics.

**Variable**	**All** **(*n =* 988)**	**Traditional uniforms**	**Sports uniforms**	**p-value**
		**(*n =* 854)**	**(*n =* 134)**	
Age (years)	11.8 ± 1.2	11.8 ± 1.2	11.8 ± 1.2	0.893
Sex (n, %)				0.512
Boys	520 (52.6%)	453 (53.0%)	67 (50.0%)	
Girls	468 (47.4%)	401 (47.0%)	67 (50.0%)	
Weight (kg)	50.1± 12.1	50.0± 12.3	50.1± 11.0	0.387
Height (cm)	151.9 ± 9.3	151.5 ± 9.2	154.7 ± 9.2	<0.001
Peak height velocity (score)	−0.58 ± 1.2	−0.60 ± 1.2	−0.50 ± 1.3	0.397
Body mass index (z-score)	1.08 ± 1.1	1.09 ± 1.1	0.97 ± 0.96	0.204
Academic achievement	5.42 ± 0.7	5.40 ± 0.7	5.60 ± 0.7	0.022
Cognitive performance (score)	99.4 ± 8.7	99.0 ± 8.8	101.4 ± 7.6	0.003
Physical fitness (z-score)	−0.29 ± 2.8	−0.36 ± 2.8	0.12 ± 2.7	0.068

Data are presented as mean ± SD or frequency (%) according to variable features. P-value: significant differences between groups (t-test or chi-square according to variable features).

**Figure 1:**
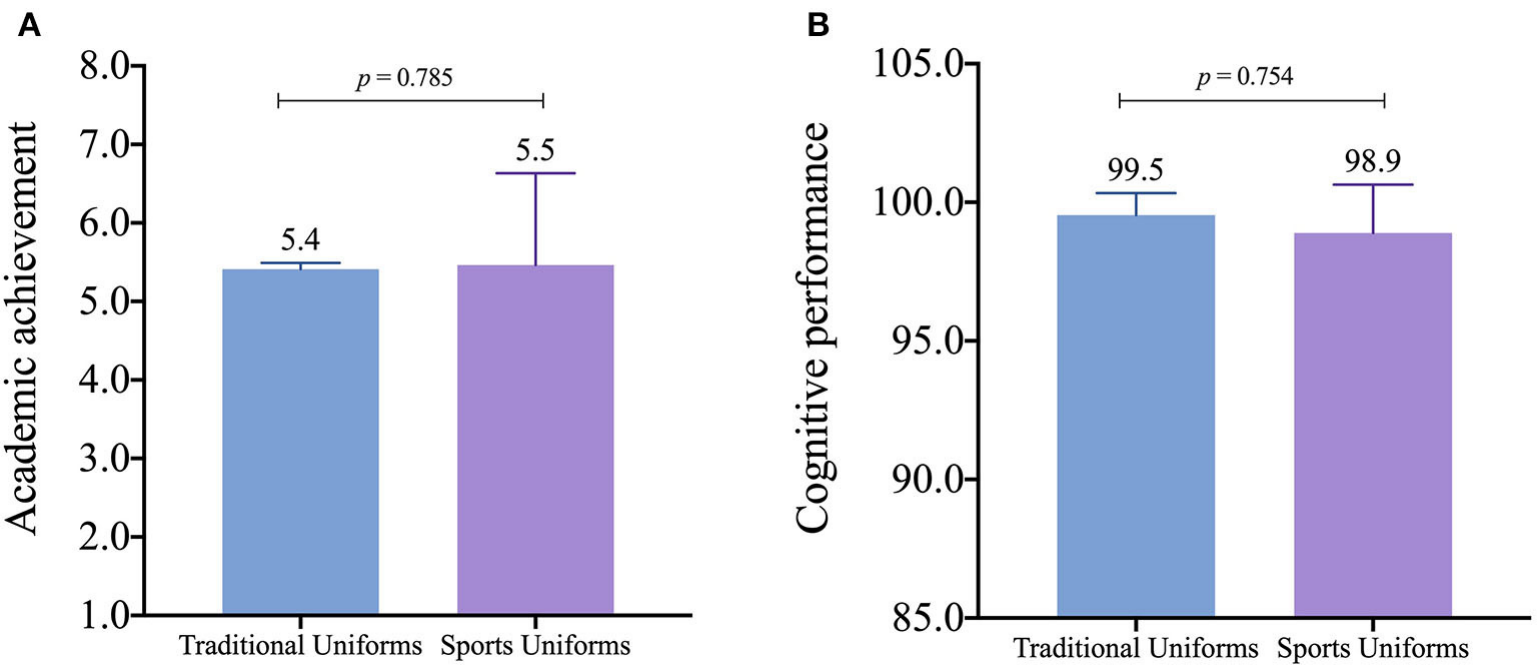
Differences between academic achievement and cognitive performance in adolescents according to uniform types. Estimated marginal means and standard error. Figure according to model 2 (adjusted for sex, PHV, BMIz, physical fitness, and SVI).

Figure 1A shows no significant differences between uniform types in academic achievement (random effect LTR was 44.0; *p* < 0.001; ICC = 0.098). Figure 1B displays no significant differences between uniform types in cognitive performance (random effect LTR was 21.7; *p* < 0.001; ICC = 0.080). Consistent results were found using model 1 and model 2 in both analyses (see Supplementary material).

**Figure 2:**
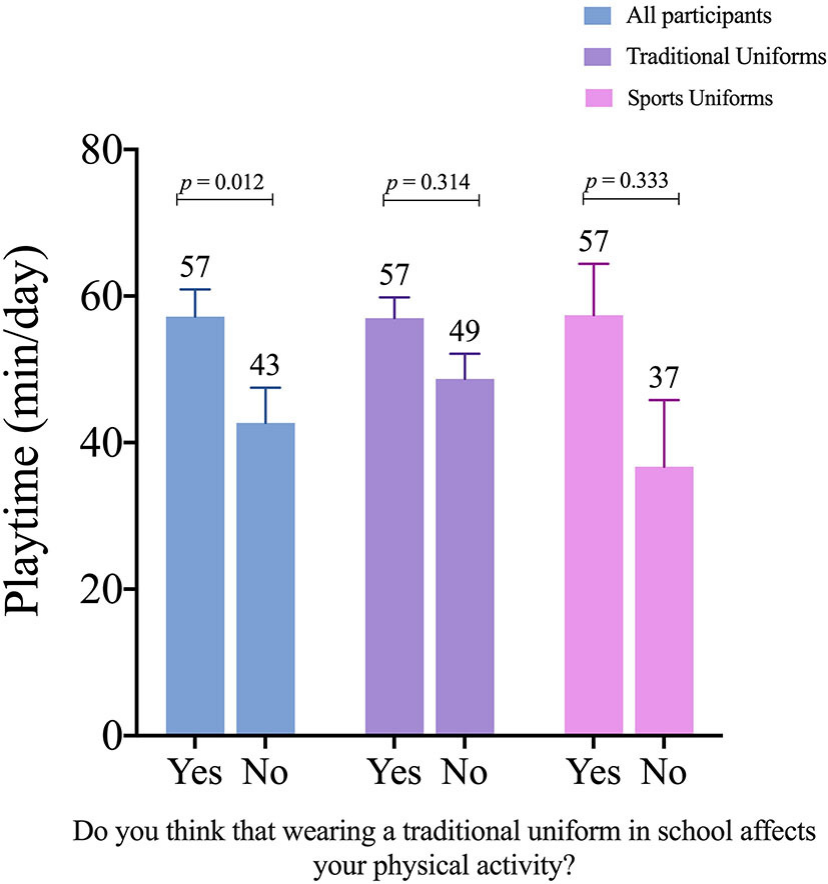
Playtime differences of adolescents according to uniform types and perception of traditional uniform affecting their physical activity. Estimated marginal means and standard error. *P*-value adjusted for multiple comparisons (Holm correction). Figure according to model 2 (adjusted for sex, PHV, BMIz, physical fitness, and SVI).

For the question, “Do you think that wearing a traditional uniform in school affects your physical activity?”, 64.1% of adolescents believe that the traditional uniform affects it (proportion test, *p* < 0.01). Those that responded affirmatively play 14.5 min more than those who respond negatively (*p* = 0.012) (see Supplementary material). Moreover, considering only those who responded affirmatively showed higher playtime than those who responded negatively (TUs: +8 min and SUs: +20 min, both *p* > 0.05, and random effect LTR was 0.11; *p* = 0.739; ICC = 0.002) (Figure 2). Consistent results were found using model 1 and model 2 (see Supplementary material).

**Figure 3:**
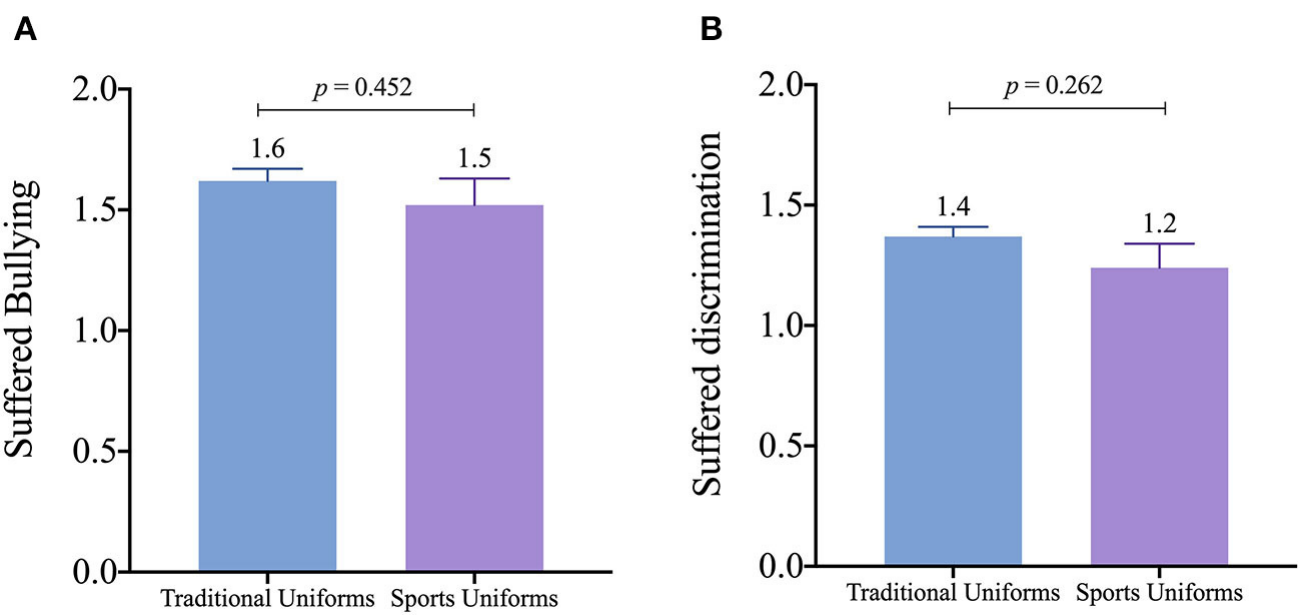
Bullying and discrimination feelings of adolescents according to uniform types. Estimated marginal means and standard error. Figure according to model 2 (adjusted for sex, PHV, BMIz, physical fitness, and SVI).

Figure 3A shows no significant differences between uniform types in feeling of bullying (random effect LTR was 8.20; *p* < 0.01; ICC = 0.026; see Supplementary material). Consistent results were found using model 1 and model 2, but there is a trend in model 1 (*p* = 0.064). While Figure 3B displays no significant discrimination differences between groups in model 2 (random effect LTR was 7.60; *p* < 0.01; ICC = 0.056), a higher discrimination feeling was found in adolescents wearing TUs in model 1 (TUs: 1.41 ± 0.05 vs. SUs: 1.13 ± 0.11, *p* = 0.041). The random effect LTR was 23.76; *p* < 0.01.

Tables 2, 3 show the proportion of responses with regard to bullying and discrimination feelings of adolescents wearing TU or SU. Significant differences in answer frequency according to bullying and discrimination feelings (χ^2^ = 10.7; *p* = 0.012 and χ^2^ = 10.3; *p* < 0.001, respectively). Lower answer frequency is observed in “usually” and “always” categories in schools wearing SUs, both bullying and discrimination.

**Table 2:** Contingency table for bullying feelings.

	**Traditional uniforms**	**Sports uniforms**	**Total**
Never	407 (54.3%)	84 (65.6%)	491 (56.0%)
Sometimes	252 (33.6%)	40 (31.3%)	292 (33.3%)
Usually	65 (8.7%)	3 (2.3%)	68 (7.8%)
Always	25 (3.3%)	1 (0.8%)	26 (3.0%)
Total	749 (100%)	128 (100%)	877 (100%)

Data are presented as frequency (% within column).

**Table 3:** Contingency table for discrimination feelings.

	**Traditional uniforms**	**Sports uniforms**	**Total**
Never	560 (74.2%)	115 (89.1%)	675 (76.4%)
Sometimes	141 (18.7%)	14 (10.9%)	155 (17.5%)
Usually	31 (4.1%)	0 (0.0%)	31 (3.5%)
Always	23 (3.0%)	0 (0.0%)	23 (2.6%)
Total	755 (100%)	129 (100%)	884 (100%)

Data are presented as frequency (% within column).

## Discussion

### Differences in academic achievement and cognitive performance

The first aim was to establish whether adolescents who wear TUs present higher academic and cognitive performance than those who wear SUs. The present findings did not find any significant differences in both educational outcomes.

In line with our findings, a Korean study that explored the link between uniforms and appearance restrictions in middle schools and high schools found no evidence that wearing school uniforms leads to better academic achievement and reported that uniforms would deny the expression of individuality and creativity (37). Concerning creativity, it is an essential cognitive resource that is fundamental in the learning process. In this line, a meta-analysis including 120 studies concludes a significantly stronger relationship between creativity and academic achievement (38); thus, wearing TUs could influence both cognitive skills and, in turn, academic achievement. Nonetheless, wearing TUs also could improve children's discipline, which was associated with higher academic performance (39).

To the best of our knowledge, this is the first study in a sample of Latin-American adolescents exploring both educational outcomes and type of uniform, and the lack of studies related to this topic makes the comparison difficult. Therefore, the present findings contribute to clarifying the scarce and divergent evidence on this matter (6). They also demonstrate that, independently of relevant covariates related to academic and cognitive performance, such as physical fitness, body composition, maturation, and school vulnerability, wearing a TU was not related to educational outcomes.

### Student's perception and differences in playtime

The second aim was to ask adolescents whether wearing a TU affects their physical activity, and their answers were compared with playtime according to the type of uniform. Two relevant findings were observed; first, 64.1% of adolescents declared that TUs affect their physical activity negatively. The second, those who responded affirmatively played 14.5 min more than those who responded negatively. Likewise, a difference of 20 min of playtime was detected in adolescents who responded positively to the question and wore SUs.

In line with the first finding, a study in an Australian primary school found similar results, where 62% of schoolchildren preferred to wear their SU every day and believe they would be more active if they could do so (40). Moreover, a study on public middle school students found that 87.3% of them dislike wearing a TU (2). Hence, the present finding from a Latin-American country confirms the opinion of Australian (40) and the United States (2) students contributing to the literature gap in this matter.

Regarding the second finding, this study highlights two main results: one with a statistical significance (playtime: 14.5 min) and the other without (playtime: 20 min). This has a justification at the public health level relevant to discuss. On the one hand, a study reported significant differences in girls' physical activity levels over boys during breaks and lunch when they wore a SU (41). In this line, girls declare that wearing TUs limits performing physical activities during school time (42). These two studies support our findings, but we do not find differences at the sex level, which suggests that both girls and boys in this group of Chilean adolescents could benefit from wearing SUs. On the other hand, adolescents who believe that the traditional uniforms affect their physical activity played 20 min more than those who do not believe so. This nonsignificant statistical difference could account for a third of the daily physical activity recommendation for children and adolescents (43). Although many physical activity interventions in schools have shown modest effects (16), small modifications are significant for getting health benefits in real contexts (16, 43).

Important to highlight is that schools' principals seem to be a key barrier related to attitudes to changing school uniform policies allowing students to wear SUs every day, but not teachers, parents, and students (40). The central concern is that the SU does not fit with ceremonial and formal activities at school. Indeed, in Chile, the school uniform must be agreed upon by principals, teachers, parents, and students (44); however, the TU is widely worn. Thus, institutional measures supported by government policies are essential to creating the conditions at the interpersonal level to promote behavioral changes in adolescents.

Therefore, the present findings contribute to the literature showing that promoting a comfortable school uniform could help to increase physical activity in adolescents. Interventional approaches are necessary to corroborate this assumption. Moreover, these findings are useful for future internal school regulations, as well as public policies at educational and health levels due to the prevalence of obesity, physical inactivity, low fitness performance, and educational achievement observed especially in schoolchildren from developing countries (45–48).

### Bullying and discrimination feelings

The third aim was to compare bullying and discrimination feelings at school according to adolescents' uniform type. In this sense, the present findings seem contrary to social belief and some studies on this matter (2, 6), showing similar feelings between groups in both indicators when the model is adjusted for SVI (model 2).

Bullying and discrimination are a global concern at the educational level. For instance, in Chile, 62% of students have reported having been victims of bullying. This percentage is one of the highest percentages based on countries assessed by the Trends in International Mathematics and Science Study-−2011 (49). One central reason is that Chile has one of the highest levels of inequality in Latin America, and its educational system has generated a high level of social segregation among the various types of schools (50, 51). These economic and social fundamentals have been associated with a greater prevalence of bullying, and, in turn, being a bully victim has been negatively correlated with academic and cognitive performance (50). However, we cannot establish causality considering bullying as the base of lower educational achievement because there is also the possibility that students who have lower performance choose to bully others (50).

In addition, a U.S. study including 6,320 children from a nationally representative sample who were followed from kindergarten entry through the end of fifth grade found that those who wore uniforms did not demonstrate better social behavior (i.e., social skills, internalizing, and externalizing behavior) (52). Relevant to note is that their findings were robust even across both public and private schools. Moreover, another study that compared students' opinions (*n* = 604) on the benefits of wearing a school uniform after implementing this measure in a public school in Nevada found a potential effect to improve the school climate and students' experiences. However, students' beliefs did not support these changes (2). A low-middle-income country (i.e., Mongolia) has reported the exclusion feelings of schoolchildren when most students wear uniforms in school (53).

Finally, and connecting this result with the previous finding addressed, the early detection of bullying and discrimination is fundamental as it could strengthen the social relationships among students and improve school coexistence, where physical activity plays a vital role at school (54). Furthermore, increased physical activity has been associated with reduced bullying victimization and enhanced cognitive performance and academic achievement (27, 55, 56). Therefore, in this large sample of Chilean adolescents, the kind of uniform wore was not related to bullying and discrimination, solving great social, family, and school principals' concerns.

### Influence of school vulnerability

Finally, it was hypothesized that possible differences in our analyses would depend mainly on the school vulnerability than the uniform type. This assumption was based on the importance and convergence of factors such as socioeconomics, inequality, and the vulnerability index at the school levels in a Latin-American sample of adolescents (20, 22, 57). Contrary to our hypothesis, model 1 and model 2 seem to be consistent and not affected by the school's vulnerability in academic achievement, cognitive performance, playtime, and bullying.

Nonetheless, discrimination was significantly affected when SVI was not present in the analysis (model 1). In other words, discrimination appreciated by students at schools seems to depend mainly on the social and economic features of the educational establishment and not on the uniform worn. In line with this finding, a study addressing the perceptions of school uniforms with socioeconomic statuses established that students of diverse socioeconomic statuses perceive school uniforms similarly (10). In addition, a multilevel study of school violence in 52 countries concluded that income inequality was the main determinant of school violence (50). Thereby, future education policies oriented to reduce bullying and discrimination might focus primarily on decreasing social gaps inside and outside the school, and in the specific case of Chile, adolescents' school uniform seems to be not a particular determinant of bullying and discrimination.

### Strength and limitations

Some strengths of this study were the large adolescent sample from a usually underrepresented region in this research area and the set of variables included (academic achievement, cognitive performance, playtime, bullying, and discrimination) that contribute to resolving a complex educational topic worldwide. Also, the cognitive performance score based on eight tasks strengthened the study measure. Furthermore, the present statistical analysis permitted control of the effect by cluster and explored a powerful indicator of school vulnerability. Finally, a large group of researchers living in Chile participated and reviewed this study.

Some study limitations were the scarce evidence comparing TUs and SUs and the non-assumption of causality due to the data characteristics. Playtime was evaluated by a self-reported question and not by objective measurement (i.e., accelerometers). Despite the large sample size of adolescents, it is not representative of Chile; hence extrapolations should be made with caution. Finally, the number of schools in each group (traditional or sports uniforms) was different, and schools, where adolescents wear SUs, do not include any public establishment. In this sense, it is relevant to mention that school type (i.e., administration) has been associated with academic achievements, which could—to a certain extent—favor schools wearing SUs. In this study, SVI shares variance with the school administration (*r* = 0.480; *p* < 0.01); nonetheless, SVI is a potent indicator that includes not only the school administration but also other relevant indicators (detailed description in the “Covariates” and “Methods” sections), being highly related to several variables used in this study (16, 28, 53).

## Conclusion

Based on the present findings and scholarly Chilean context, wearing a TU seems to have no crucial educational advantage at an academic and cognitive level compared with SUs. Therefore, it could be recommended that the school community consider adolescents' opinions and increase instances for promoting a uniform that will be more comfortable such as SUs, which could permit them to increase their physical activity during the school day. Additionally, this feasible and low-cost measure would not be related to higher feelings of bullying and discrimination.

## Data availability statement

The datasets presented in this article are not readily available because ethical restrictions. Requests to access the datasets should be directed to carlos.cristi.montero@gmail.com.

## Ethics statement

The studies involving human participants were reviewed and approved by Bioethics and Biosafety Committee of the Ethics Committee of Pontificia Universidad Católica de Valparaíso (BIOEPUCV-H103–2016). Written informed consent to participate in this study was provided by the participants' legal guardian/next of kin.

## Author contributions

CC-M conceived and designed the data analysis and manuscript. CC-M was responsible for coordinating the study, acquiring the data, and writing the first manuscript version. All authors contributed significantly to editing the manuscript and agreed to the final version.

## Funding

CC-M received funding for the Cogni-Action Project from the National Commission for Scientific and Technological Research CONICYT/FONDECYT INICIACION 2016 grant No. 11160703 (Chile).

## Conflict of interest

The authors declare that the research was conducted in the absence of any commercial or financial relationships that could be construed as a potential conflict of interest.

## Publisher's note

All claims expressed in this article are solely those of the authors and do not necessarily represent those of their affiliated organizations, or those of the publisher, the editors and the reviewers. Any product that may be evaluated in this article, or claim that may be made by its manufacturer, is not guaranteed or endorsed by the publisher.
