# Supplementary material for: Which one? A comparative study of traditional and sports uniforms on academic achievement, cognitive performance, playtime, bullying, and discrimination in adolescents: The Cogni-Action Project
Source: Front Public Health. 2022 Aug 12;10:917970. doi: 10.3389/fpubh.2022.917970 (PMC9411804; doi:10.3389/fpubh.2022.917970)
Supplement: Supplementary file 1 [file Data_Sheet_1.docx]

**Supplementary material**

**Table 1.** Fixed effects parameter estimates for academic achievement.

Model 1: Without SVI

|  |  |  | **95% Confidence Interval** | |  |  |  |
| --- | --- | --- | --- | --- | --- | --- | --- |
| **Names** | **Estimate** | **SE** | **Lower** | **Upper** | **df** | **t** | **p** |
| (Intercept) | 5.438 | 0.079 | 5.283 | 5.594 | 13.321 | 68.549 | < .001 |
| Uniform type | 0.088 | 0.159 | -0.223 | 0.399 | 13.299 | 0.553 | 0.589 |
| PHV | -0.097 | 0.023 | -0.141 | -0.053 | 981.996 | -4.294 | < .001 |
| BMI | -0.051 | 0.023 | -0.097 | -0.006 | 973.990 | -2.211 | 0.027 |
| Fitness | 0.015 | 0.009 | -0.003 | 0.033 | 981.276 | 1.626 | 0.104 |
| Sex | 0.142 | 0.027 | 0.088 | 0.195 | 981.102 | 5.212 | < .001 |

LTR: 44.2; p-value <0.001; Estimate (B values); SE: standard error; df: degrees of freedom.

Model 2: With SVI

|  |  |  | **95% Confidence Interval** | |  |  |  |
| --- | --- | --- | --- | --- | --- | --- | --- |
| **Names** | **Estimate** | **SE** | **Lower** | **Upper** | **df** | **t** | **p** |
| (Intercept) | 5.428 | 0.087 | 5.258 | 5.598 | 11.912 | 62.408 | < .001 |
| Uniform type | 0.053 | 0.192 | -0.322 | 0.429 | 10.915 | 0.279 | 0.785 |
| PHV | -0.097 | 0.023 | -0.141 | -0.053 | 981.000 | -4.287 | < .001 |
| BMI | -0.051 | 0.023 | -0.097 | -0.006 | 973.575 | -2.202 | 0.028 |
| Fitness | 0.015 | 0.009 | -0.003 | 0.033 | 980.573 | 1.626 | 0.104 |
| Sex | 0.141 | 0.027 | 0.088 | 0.195 | 980.056 | 5.203 | < .001 |
| SVI | -0.024 | 0.071 | -0.163 | 0.116 | 10.910 | -0.334 | 0.744 |

LTR: 44.0; p-value <0.001; Estimate (B values); SE: standard error; df: degrees of freedom.

**Table 2.** Fixed effects parameter estimates for cognitive performance.

Model 1: Without SVI

|  |  |  | **95% Confidence Interval** | |  |  |  |
| --- | --- | --- | --- | --- | --- | --- | --- |
| **Names** | **Estimate** | **SE** | **Lower** | **Upper** | **df** | **t** | **p** |
| (Intercept) | 100.158 | 1.135 | 97.934 | 102.382 | 13.275 | 88.260 | < .001 |
| Uniform type | 2.664 | 2.269 | -1.782 | 7.110 | 13.256 | 1.174 | 0.261 |
| PHV | 0.025 | 0.261 | -0.486 | 0.536 | 980.256 | 0.096 | 0.923 |
| BMI | -0.001 | 0.267 | -0.524 | 0.522 | 972.545 | -0.003 | 0.997 |
| Fitness | 0.455 | 0.106 | 0.248 | 0.661 | 981.477 | 4.307 | < .001 |
| Sex | -0.023 | 0.313 | -0.636 | 0.590 | 978.535 | -0.073 | 0.942 |

LTR: 95.1; p-value <0.001; Estimate (B values); SE: standard error; df: degrees of freedom.

Model 2: With SVI

|  |  |  | **95% Confidence Interval** | |  |  |  |
| --- | --- | --- | --- | --- | --- | --- | --- |
| **Names** | **Estimate** | **SE** | **Lower** | **Upper** | **df** | **t** | **p** |
| (Intercept) | 99.219 | 0.915 | 97.427 | 101.012 | 10.677 | 108.478 | < .001 |
| Uniform type | -0.644 | 2.006 | -4.576 | 3.288 | 9.668 | -0.321 | 0.755 |
| PHV | 0.050 | 0.260 | -0.460 | 0.559 | 980.497 | 0.191 | 0.848 |
| BMI | 0.001 | 0.267 | -0.522 | 0.524 | 973.452 | 0.003 | 0.998 |
| Fitness | 0.451 | 0.105 | 0.244 | 0.657 | 978.616 | 4.283 | < .001 |
| Sex | -0.070 | 0.312 | -0.682 | 0.543 | 980.712 | -0.223 | 0.824 |
| SVI | -2.343 | 0.744 | -3.801 | -0.885 | 9.625 | -3.149 | 0.011 |

LTR: 21.7; p-value <0.001; Estimate (B values); SE: standard error; df: degrees of freedom.

**Table 3.** Fixed effects parameter estimates for playtime by answer.

Model 1: Without SVI

|  |  |  | **95% Confidence Interval** | |  |  |  |
| --- | --- | --- | --- | --- | --- | --- | --- |
| **Names** | **Estimate** | **SE** | **Lower** | **Upper** | **df** | **t** | **p** |
| (Intercept) | 51.533 | 2.827 | 45.993 | 57.073 | 981.000 | 18.231 | < .001 |
| Uniform type | -2.574 | 5.577 | -13.505 | 8.357 | 981.000 | -0.461 | 0.645 |
| PHV | -3.116 | 1.891 | -6.823 | 0.591 | 981.000 | -1.648 | 0.100 |
| BMI | 0.193 | 1.992 | -3.711 | 4.097 | 981.000 | 0.097 | 0.923 |
| Fitness | 2.506 | 0.758 | 1.020 | 3.992 | 981.000 | 3.305 | < .001 |
| Sex | -9.237 | 2.314 | -13.773 | -4.701 | 981.000 | -3.991 | < .001 |
| Uniform perception | 10.041 | 3.955 | 2.288 | 17.793 | 981.000 | 2.538 | 0.011 |

 LTR: 0.0; p-value = 1.000; Estimate (B values); SE: standard error; df: degrees of freedom.

Model 2: With SVI

|  |  |  | **95% Confidence Interval** | |  |  |  |
| --- | --- | --- | --- | --- | --- | --- | --- |
| **Names** | **Estimate** | **SE** | **Lower** | **Upper** | **df** | **t** | **p** |
| (Intercept) | 51.103 | 3.072 | 45.082 | 57.124 | 18.270 | 16.635 | < .001 |
| Uniform type | -3.996 | 6.281 | -16.306 | 8.314 | 15.283 | -0.636 | 0.534 |
| PHV | -3.185 | 1.898 | -6.904 | 0.535 | 837.687 | -1.678 | 0.094 |
| BMI | 0.211 | 1.994 | -3.696 | 4.118 | 979.907 | 0.106 | 0.916 |
| Fitness | 2.441 | 0.764 | 0.943 | 3.938 | 750.688 | 3.195 | 0.001 |
| Sex | -9.266 | 2.319 | -13.811 | -4.720 | 949.056 | -3.995 | < .001 |
| Uniform perception | 10.050 | 3.962 | 2.285 | 17.816 | 944.224 | 2.537 | 0.011 |
| SVI | -1.310 | 2.203 | -5.628 | 3.007 | 9.471 | -0.595 | 0.566 |

LTR: 0.1; p-value = 0.767; Estimate (B values); SE: standard error; df: degrees of freedom.

**Table 4.** Post Hoc Comparisons

**School Uniform Type ✻ Perception Uniform Affect Physical Activity**

| **Uniform** | **Perception** | **Uniform** | **Perception** | **Differences** | **SE** | **t** | **df** | **P_Bonf_** |
| --- | --- | --- | --- | --- | --- | --- | --- | --- |
| Sport | No | Sport | Yes | -20.8 | 10.8 | -1.92 | 930 | 0.333 |
| Traditional | No | Traditional | Yes | -8.4 | 4.3 | -1.94 | 946 | 0.314 |

**Perception Uniform Affect Physical Activity**

| **Perception** | **Perception** | **Differences** | **SE** | **t** | **df** | **P_Bonf_** |
| --- | --- | --- | --- | --- | --- | --- |
| No affect | Yes affect | -14.6 | 5.8 | -2.5 | 929 | 0.012 |

**Table 5.** Fixed effects parameter estimates for bullying.

Model 1: Without SVI

|  |  |  | **95% Confidence Interval** | |  |  |  |
| --- | --- | --- | --- | --- | --- | --- | --- |
| **Names** | **Estimate** | **SE** | **Lower** | **Upper** | **df** | **t** | **p** |
| (Intercept) | 1.523 | 0.067 | 1.392 | 1.654 | 14.542 | 22.846 | < .001 |
| Uniform type | -0.270 | 0.133 | -0.532 | -0.009 | 14.544 | -2.030 | 0.061 |
| PHV | -0.093 | 0.025 | -0.143 | -0.044 | 868.575 | -3.705 | < .001 |
| BMI | 0.022 | 0.026 | -0.029 | 0.072 | 866.300 | 0.841 | 0.400 |
| Fitness | -0.011 | 0.010 | -0.031 | 0.009 | 856.843 | -1.115 | 0.265 |
| Sex | 0.051 | 0.030 | -0.009 | 0.110 | 870.632 | 1.677 | 0.094 |

LTR: 23.5; p-value <0.001; Estimate (B values); SE: standard error; df: degrees of freedom.

Model 2: With SVI

|  |  |  | **95% Confidence Interval** | |  |  |  |
| --- | --- | --- | --- | --- | --- | --- | --- |
| **Names** | **Estimate** | **SE** | **Lower** | **Upper** | **df** | **t** | **p** |
| (Intercept) | 1.562 | 0.056 | 1.453 | 1.672 | 13.717 | 27.915 | < .001 |
| Uniform type | -0.100 | 0.120 | -0.336 | 0.135 | 11.540 | -0.835 | 0.421 |
| PHV | -0.092 | 0.025 | -0.141 | -0.043 | 854.464 | -3.676 | < .001 |
| BMI | 0.022 | 0.026 | -0.028 | 0.072 | 867.653 | 0.851 | 0.395 |
| Fitness | -0.009 | 0.010 | -0.028 | 0.011 | 826.450 | -0.883 | 0.378 |
| Sex | 0.053 | 0.030 | -0.006 | 0.112 | 865.103 | 1.758 | 0.079 |
| SVI | 0.130 | 0.045 | 0.042 | 0.217 | 10.929 | 2.913 | 0.014 |

LTR: 8.2; p-value = 0.014; Estimate (B values); SE: standard error; df: degrees of freedom.

**Table 6.** Fixed effects parameter estimates for discrimination.

Model 1: Without SVI

|  |  |  | **95% Confidence Interval** | |  |  |  |
| --- | --- | --- | --- | --- | --- | --- | --- |
| **Names** | **Estimate** | **SE** | **Lower** | **Upper** | **df** | **t** | **p** |
| (Intercept) | 1.266 | 0.060 | 1.148 | 1.385 | 13.836 | 20.938 | < .001 |
| Uniform type | -0.274 | 0.121 | -0.511 | -0.037 | 13.832 | -2.265 | 0.040 |
| PHV | -0.072 | 0.022 | -0.115 | -0.029 | 875.862 | -3.264 | 0.001 |
| BMI | 0.045 | 0.022 | 0.001 | 0.089 | 872.938 | 2.023 | 0.043 |
| Fitness | -0.008 | 0.009 | -0.025 | 0.010 | 865.047 | -0.863 | 0.388 |
| Sex | 0.041 | 0.026 | -0.011 | 0.093 | 877.865 | 1.551 | 0.121 |

LTR: 23.8; p-value < 0.001; Estimate (B values); SE: standard error; df: degrees of freedom.

Model 2: With SVI

|  |  |  | **95% Confidence Interval** | |  |  |  |
| --- | --- | --- | --- | --- | --- | --- | --- |
|  | **Estimate** | **SE** | **Lower** | **Upper** | **df** | **t** | **p** |
| (Intercept) | 1.298 | 0.052 | 1.197 | 1.399 | 11.401 | 25.175 | < .001 |
| Uniform type | -0.132 | 0.111 | -0.350 | 0.086 | 9.647 | -1.189 | 0.263 |
| PHV | -0.071 | 0.022 | -0.114 | -0.028 | 860.803 | -3.262 | 0.001 |
| BMI | 0.045 | 0.022 | 0.001 | 0.089 | 873.788 | 2.020 | 0.044 |
| Fitness | -0.006 | 0.009 | -0.023 | 0.011 | 832.263 | -0.702 | 0.483 |
| Sex | 0.043 | 0.026 | -0.009 | 0.094 | 873.036 | 1.614 | 0.107 |
| SVI | 0.109 | 0.041 | 0.028 | 0.190 | 9.274 | 2.650 | 0.026 |

LTR: 7.6; p-value = 0.006; Estimate (B values); SE: standard error; df: degrees of freedom.
